# Decreased femoral head–neck offset: a possible risk factor for ACL injury

**DOI:** 10.1007/s00167-012-1881-1

**Published:** 2012-01-28

**Authors:** Marc Philippon, Christopher Dewing, Karen Briggs, J. Richard Steadman

**Affiliations:** 1 Steadman Philippon Research Institute, Clinical Research, 181 W. Meadow Drive, Suite 1000, Vail, CO 81657 USA; 2 Naval Medical Center, San Diego, CA USA

**Keywords:** ACL injuries, Femoroacetabular impingement, Alpha angle, Femoral head–neck offset

## Abstract

**Purpose:**

Reduction in anterior cruciate ligament (ACL) injuries in young, active individuals continues to be a major goal in sports medicine. The purpose of this study was to determine the head–neck offset, as measured by AP pelvis alpha angles, in patients presenting to a single surgeon with isolated ACL and non-ACL knee injuries.

**Methods:**

In a group of 48 patients with complete, primary ACL rupture and 42 controls with non-ACL injury (i.e., meniscus tear, cartilage defect), a single surgeon, blinded to the diagnosis, took radiographic measures of the AP alpha angle of both hips and the weight-bearing line at both knees. All knee pathology was confirmed with knee arthroscopy. Inclusion criteria included no previous hip or knee surgery, and long-leg standing alignment radiographic series completed at index visit.

**Results:**

There was no difference in gender distribution, height, BMI or age between groups. ACL-injured patients had a significantly higher alpha angle (mean = 84, SD = 14) on the injured side than the controls (mean = 59, SD = 7, *p* < 0.0001). Ninety-four percent of the ACL-injured group had alpha angles over 60°, while only 35% of the non-ACL-injured group had alpha angles over 60° (*p* = 0.001). Those patients with alpha angle over 60° were 27 times more likely (95% CI 6.4–131) to be in the ACL injury group than those patients with alpha angle 60° or less (*p* = 0.001).

**Conclusion:**

Our findings establish an important preliminary correlation between ACL injury and diminished femoral head–neck offset, as characterized by abnormal, elevated alpha angles.

**Level of evidence:**

Prognostic study, Level III.

## Introduction

Reduction in anterior cruciate ligament (ACL) injuries in young active individuals continues to be a major goal in sports medicine. Recent research has highlighted the interaction between altered hip biomechanics and knee injury patterns [3, 5, 12, 14, 15, 18, 22]. Recently, video analysis of ACL injuries in athletes has shown consistent patterns of valgus loading of the knee near full extension with internal or external rotation [18]. Higher hip flexion angles at impact, but no differences in hip abduction angles have also been associated with ACL injury on video analysis [3]. Assessment of hip rotation by video analysis is impractical; however, in an effort to develop injury prevention programs, a better understanding of the interaction between the hip and knee in the ACL-injured population is critical.

Femoroacetabular impingement has been identified more frequently in the active population [2, 4, 6, 11, 13, 19–21]. Bony abnormalities around the femoral head cause cam impingement, while acetabular bony abnormalities cause pincer impingement [7]. The alpha angle is commonly used as a measure of cam impingement [13, 17]. In addition to a large alpha angle, decreased range of motion has also been described with cam impingement [2, 6, 11, 13, 19–21, 23]. Recently, investigators have seen similar decreases in hip motion in individuals who have suffered an ACL injury. In 2008, investigators measured hip range of motion in 50 soccer players who had sustained a non-contact ACL injury [9]. This study showed a strong association between hip range of motion and the presence of a non-contact ACL injury. In these soccer players, the main reason for the loss of motion was decreased internal rotation [9].

The purpose of this study was to determine the head–neck offset, as measured by AP pelvis alpha angles, in patients presenting to a single surgeon with isolated ACL and non-ACL knee injuries. The hypothesis was that patients who presented with acute ACL injury would demonstrate diminished femoral head–neck offset, by exhibiting an increased alpha angle, when compared to patients with non-ACL knee injuries.

## Materials and methods

A retrospective review of a prospectively collected database identified 50 consecutive patients with primary ACL rupture and 50 consecutive patients with non-ACL injury (i.e., meniscus tear, cartilage defect). All knee pathology was confirmed with knee arthroscopy by a single surgeon. Inclusion criteria included no previous hip or knee surgery, and complete long-leg standing alignment radiographic series completed at index visit. Forty-eight of the 50 initially identified patients with primary ACL rupture and 42 of the patient with non-ACL injury met the inclusion criteria.

**Fig. 1:**
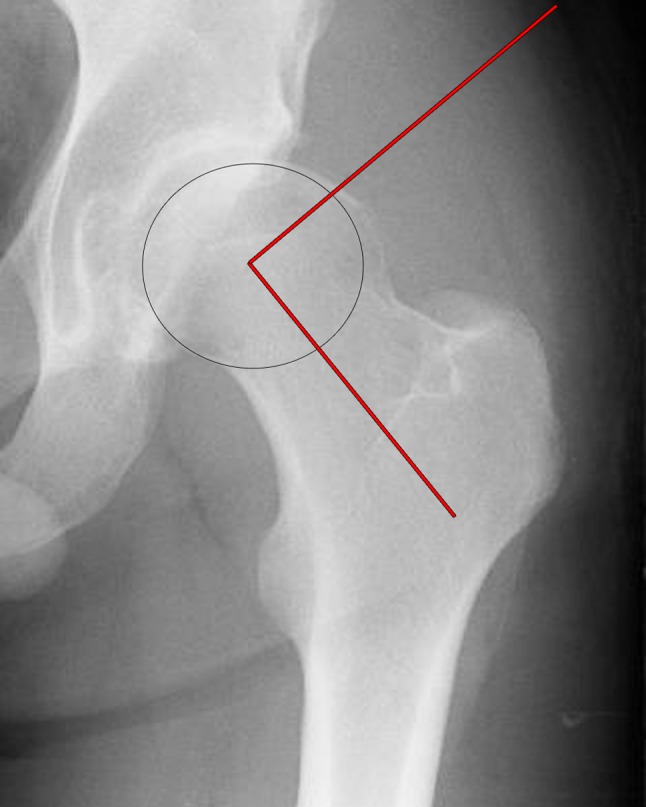
A magnified anterior posterior radiograph view taken from a pelvis radiograph with an increased alpha angle. The alpha angle subtended between a line from the midline of the femur to the center of the femoral head and a line from the center of the femoral head to the point at which the femoral head deviated from a circular template overlay

A single hip surgeon with experience in hip pathomorphology, blinded to the diagnosis, took radiographic measures of the AP alpha angle of both hips and the weight-bearing line at both knees in all patients. The alpha angle, which is commonly used, was described by Notzli et al. [17] and uses the tilted axial scans passing through the center of the head of the MRI which is equivalent to the lateral view on radiographs. Since all patients in this study presented with knee injuries, the AP long-standing radiograph was the only series available with both hips. Using the method described by Gosvig et al. [10], the center of the femoral head was found, and a line was drawn from the center of the head along the middle of the femoral neck. With the center of the circle equal to the center of the femoral head, a circle was drawn around the circumference of the femoral head. Starting from the first point where any bone deviated from outside this circle, a line was drawn to the center of the femoral head. This is the point where the bony abnormality increased the radius of the circle. The angle between the middle of the femoral neck and the point of increase is the AP alpha angle (Fig. 1). These measurements were taken with a digital goniometer by an orthopedic surgeon who completed a fellowship in hip arthroscopy (OfficePACS, Stryker Imaging, Flower Mound, TX, USA). The precision of the measurement tool was 0.5°. Previous studies have shown that the alpha angle demonstrated excellent intra-tester reliability in an experienced observer [13]. For this study, an abnormal alpha angle was operationally defined as greater than 60°.

The weight-bearing line was measured as previously described [4]. The weight-bearing line was determined by drawing a line from the center of the femoral head to the center of the tibial plafond. The width of the tibial plateau was then measured. The distance from the medial edge of the tibial plateau to the weight-bearing line was divided by the width of the tibial plateau. This provided a percentage. The medial aspect of the tibial plateau was defined as 0%, and the lateral aspect was defined as 100%.

### Statistical analysis

In addition, demographic data, surgical data and the mechanism of injury were also collected. A pre-hoc power analysis was performed to determine the number of subjects needed for the study. On the basis of an effect size of 0.5 and 95% power, 42 patients were needed (G*Power V 3.1.2, Universitat Kiel, Germany). Comparison of continuous variables (age, percent alignment, alpha angle) with binary categorical variables was made using the independent samples t test. Comparison of continuous variables was made using the Pearson correlation coefficient. Age, percent alignment and alpha angle were all normally distributed (*p* > 0.05). All reported *p* values were 2-tailed with a level of 0.05, indicating statistical significance. Statistical analysis was performed using SPSS (version 11, SPSS Inc, Chicago, IL, USA) software package. This investigation was approved by an institutional review board, and all data were collected in conformity with its regulations.

## Results

There was no difference in gender distribution, height, BMI or age between groups (Table 1). Knee injuries confirmed at arthroscopy are described in Table 2.

**Table 1:** Demographics of each study group

	Non-ACL injury	ACL injury	*p* value
*N*	42	48	
Age	31.5 (17–60)	32.5 (17–60)	n.s.
Male/female	26:16	32:16	n.s.
BMI	24.3 (SD = 4)	24.3 (SD = 3)	n.s.
Height (cm)	175 (SD = 10)	174 (SD = 9)	n.s.

**Table 2:** Arthroscopic findings at knee arthroscopy in both groups

	ACL injury (%)	Non-ACL injury (%)
ACL	100	0
Meniscus	58	33
Cartilage defect	42	7
Plica	56	78
Synovectomy	15	33
Loose bodies	8	10

There was no significant difference between ACL-injured patients and controls in terms of the weight-bearing line at the knee. The ACL-injured group had an average percent deviation of 39% (SD = 12%), and the control group had an average percent deviation of 43% (SD = 15%, *p* = 0.230) on the operative knee. On the non-operative knee, there was also no difference between the ACL-injured group (38%, SD = 13%) and the control group (42%, SD = 15%) (n.s.). Alignment fell within the middle half of the joint (25–75%) in 85% of the operative knees and 85% of the non-operative knees. For all patients, there was a correlation between the operative knee alignment percentage and the non-operative knee alignment percentage (*r* = 0.701; *p* = 0.0001). This was also true when compared within each group.

**Fig. 2:**
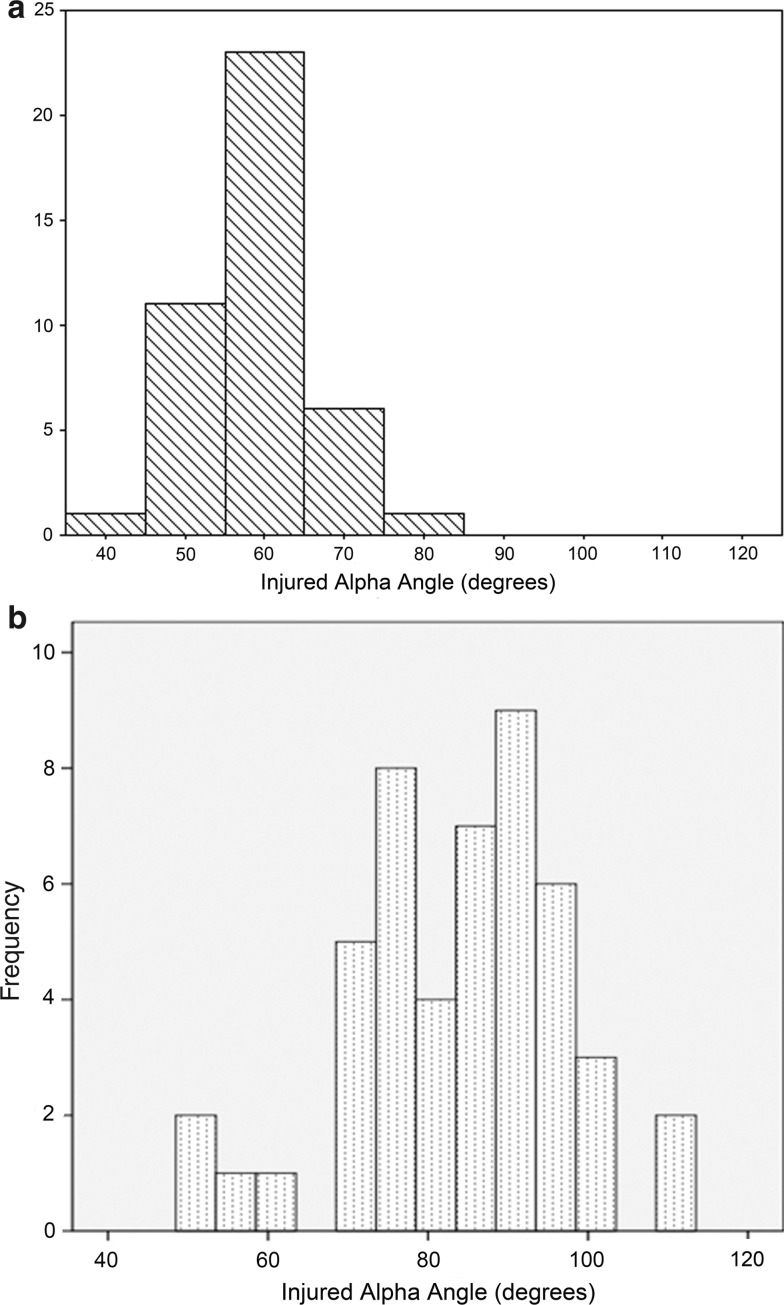
**a** Distribution of the alpha angles in the non-ACL-injured group. The mean is 59, and the distribution peaks around 60°. This is compared to (**b**) which shows the distribution of the ACL-injured group. The mean is 84 and the distribution peaks around 90, showing a marked shift in the distribution compared to the non-ACL-injured group

ACL-injured patients had a significantly higher alpha angle compared to the control group on the operative knee side (*p* < 0.01) (Fig. 2; Table 3). Ninety-four percent of the ACL-injured group had alpha angles over 60°, while only 35% of the non-ACL-injured group had alpha angles over 60° (*p* = 0.001). Those patients with alpha angle over 60° were 27 times more likely (95% CI 6.4–131) to be in the ACL injury group than those patients with alpha angle 60° or less (*p* = 0.001).

**Table 3:** Comparison of alpha angles between groups

	ACL-injured group	Non-ACL-injured group	*p* value*
Alpha angle on operative knee side	84° SD = 13	Mean = 59° SD = 7	*p* < 0.01
Alpha angle on non-operative knee side	77° SD = 17	Mean = 60° SD = 9	*p* < 0.01
*p* value**	*p* = 0.007	n.s.	

* Comparison between ACL-injured group and non-ACL-injured group

** Comparison between operative knee side and non-operative knee side

In the control group, alpha angle correlated with patient age (*r* = 0.379; *p* = 0.013) and operative side correlated with the non-operative side alpha angle (*r* = 0.391; *p* = 0.011). In the ACL-injured group, alpha angle did not significantly correlate with patient age (*r* = 0.150; n.s) but operative side alpha angle did correlate with the non-operative side alpha angle (*r* = 0.414; *p* = 0.0001).

In the control group, the mean alpha angle for males was 60 and the mean alpha angle for females was 57 (n.s.). In the ACL-injured group, the average alpha angle for males was 87 and the average alpha angles for females was 79 (*p* = 0.042). Female patients with alpha angle over 60° were 15 times more likely (95% CI 2.5–95) to be in the ACL injury group than female patients with alpha angle 60° or less (*p* = 0.001). Male patients with alpha angle over 60° were 49.6 times more likely (95% CI 5.8–422) to be in the ACL injury group than male patients with alpha angle 60° or less (*p* = 0.001).

## Discussion

The most important finding in the study was that patients who had ACL knee injuries had higher hip alpha angles compared to patients with non-ACL knee injuries. The hypothesis was confirmed in this study. Patients with an alpha angle greater than 60° were at increased odds of having an ACL injury. These increased odds were seen in both males and females; however, the odds were higher in males.

While multiple studies have described the alpha angle of the hip obtained from the radial MRI in line with the femoral neck, such measures remain impractical for incidence studies. Recent work has demonstrated that the AP view, if anything, underestimates the alpha angle. Gosvig et al. [10] evaluated over 2400 radiographic hip series and showed a close agreement between alpha angles, as measured by AP versus cross-table lateral view in 164 randomly selected patients. They determined gender-specific mean alpha angle values for AP measurements, reporting normal values at <68 for males and <50 for females and pathologic levels at >83 for males and >57 for females [9]. In the present study, mean alpha angle of 60° in the male non-ACL-injured cohort and 57° in the female non-ACL-injured cohort fall within the reported normal angles as determined by Gosvig et al. for males, but is higher for females. While no set alpha angle measurement has been agreed upon as a definition of abnormal head–neck offset, for this study, abnormal was operationally defined as greater than 60°. Males in the ACL injury cohort had mean alpha angle of 86 and the females had a mean alpha angle of 79, which are markedly higher than previously reported limits of normal. The strong correlation for both genders between abnormally elevated alpha angles and primary ACL injury suggests a possible relationship between altered hip biomechanics and ACL injury.

There is consensus among leading researchers that alterations in the kinetic chain of the trunk, hip, knee, ankle and foot contribute to ACL injury [12]. The extent that altered biomechanics of each joint contributes to injury pattern has yet to be determined. Recent jump and landing studies examining the biomechanical relationship between hip positioning and muscular fatigue in relation to knee kinematics have improved our understanding of the possible interactions between hip biomechanics and ACL injury [14, 15].

A comprehensive biomechanical theory to explain our findings of abnormally elevated alpha angles in our ACL-injured cohort is beyond the limits of this paper. Previous research has demonstrated progressive loss of internal rotation of the hip with increasing alpha angles [13]. The observed loss of internal rotation may be attributed both to bony impingement from decreased offset and to adaptive changes in soft tissue and muscle/tendon balance about the hip. Patients with abnormally elevated alpha angles may have diminished capacity at the hip to accommodate overall lower extremity internal rotation moments, potentially exposing the knee and the ACL to greater rotational stresses. In addition, another study has shown that improving the femoral head–neck offset may improve the range of motion in the hip, specifically flexion [8].

Limitations of this study include the retrospective nature, although every attempt was made to carefully match the age and gender of our test and control groups. Only cam impingement was evaluated on radiographs and did not evaluate pincer impingement. Measurements of hip rotation in this series of patients were also not available. The clinical impact of the observed correlation between ACL injury and abnormal alpha angle would be significantly stronger if it was shown that ACL patients had a significantly diminished arc of hip rotation, as compared to controls. Another limitation is the relatively small sample size and the assessment of the alpha angle. The assessments of femoral head/neck asphericity may be limited by our measures calculated by AP radiographs only. While the AP view may underestimate the alpha angle, the addition of a lateral view, such as the Dunn lateral view, has been shown to be more sensitive and correlate better with axial MRI in some studies [1, 16]. Although this limits the study, a significant relationship between alpha angle and ACL injury was shown. This information may help identify patients, who present with hip pain, who may benefit from ACL prevention programs due to increased risk of ACL injury. More research is needed to provide sufficient evidence to include this in the clinical treatment algorithm.

## Conclusions

This study showed correlation between ACL injury and diminished femoral head–neck offset, as characterized by abnormal, elevated alpha angles. Further work is needed to determine the extent of how cam-type femoroacetabular impingement of the hip alters lower extremity biomechanics, potentially predisposing patients to specific knee injury patterns. More refined understanding of these interactions may ultimately create the opportunity to improve the effectiveness of ACL injury prevention programs.
